# Prognostic and clinicopathological significance of long noncoding RNA HOXA11-AS expression in human solid tumors: a meta-analysis

**DOI:** 10.1186/s12935-017-0498-3

**Published:** 2018-01-03

**Authors:** Shidai Mu, Lisha Ai, Fengjuan Fan, Chunyan Sun, Yu Hu

**Affiliations:** Institute of Hematology, Union Hospital, Tongji Medical College, Huazhong University of Science and Technology, Wuhan, 430022 China

**Keywords:** Long noncoding RNA, HOXA11-AS, Prognosis, Meta-analysis

## Abstract

**Background:**

Recent studies have emphasized the important prognostic role of long noncoding RNAs (lncRNAs) in various types of cancers. Here we conducted a meta-analysis to investigate whether lncRNA HOXA11-AS can be served as a prognostic biomarker in human cancers.

**Patients/methods:**

We systematically searched PubMed, Embase, ISI Web of Science, and SCOPUS for relevant studies, to investigate the prognostic significance of HOXA11-AS expression in cancer patients. Odds ratios (ORs) or hazards ratios (HRs) with corresponding 95% confidence intervals (CIs) are pooled to estimate the association between HOXA11-AS expression and clinicopathological parameters or survival of cancer patients.

**Results:**

A total of eight eligible studies with 1320 cancer patients were enrolled in our meta-analysis. The results revealed that increased expression level of HOXA11-AS was significantly associated with clinicopathological parameters including more lymph node metastasis (OR = 2.06, 95% CI 1.31–3.25), advanced tumor stage (OR = 4.22, 95% CI 2.60–6.85), as well as poor tumor differentiation (OR = 2.49, 95 CI 1.47–4.20), but not correlated with age (*p* = 0.101) or gender (*p* = 0.845). In addition, cancer patients with high HOXA11-AS are prognosed to have shorter OS (pooled HR = 1.86, 95% CI 1.39–2.48) and PFS (pooled HR = 2.47, 95% CI 1.29–4.75).

**Conclusions:**

HOXA11-AS overexpression might be a convinced unfavorable prognostic factor that helps the clinical decision-making process.

## Background

Although the surgical techniques and chemotherapy/radiotherapy regimens are greatly improved, cancer is still a serious worldwide public health issue and is the second leading cause of death in the United States [1]. In 2015, 4,292,000 new cancer cases and 2,814,000 cancer deaths are projected to occur in China [2]. Therefore, identifying novel biomarkers for early diagnosis and prognosis is necessary for a better control of cancer.

High-throughput genomic platforms revealed that numerous sites within the human genome are transcribed into noncoding transcripts (ncRNAs), which have recently emerged as critical regulators of gene expression. Long noncoding RNAs (lncRNAs) are a class of ncRNAs longer than 200 nucleotides without protein-coding capacity [3]. Several studies have shown that dysregulated lncRNA expression in various types of cancers is involved in tumorigenesis and cancer metastasis through silencing tumor suppressors or activation of oncogenes at epigenetic, transcriptional and post-transcriptional levels [4–8]. LncRNAs have been implicated as promising biomarkers for diagnosis and prognosis in several cancers [9–11].

HOXA11-AS, the homeobox A11 antisense lncRNA, is located near the homeobox A11 (HOXA11) gene [12]. Human homeobox (HOX) gene clusters are essential for morphological development, which determine the identity of body segments. Recently studies suggested abnormal expression of HOX in various cancers. Protein coding genes are located on the sense strand of the HOXA gene clusters, while noncoding genes are located on the antisense strand [13]. The lncRNA HOXA11-AS was first identified in cervical cancer [14], and upregulated HOXA11-AS expression was closely associated with tumor progression and poor prognosis. Then the role of HOXA11-AS in cancer progression was identified in many other types of cancers, including gastric cancer [15], epithelial ovarian cancers [12], bladder cancer, cervical cancer [14] and glioma [16]. In vitro and in vivo assays evaluating the effects of HOXA11-AS alterations revealed a complex integrated phenotype affecting cell growth, apoptosis, migration, invasion and stemness maintenance through multiple biologic processes, such as epithelial–mesenchymal transition (EMT) [13, 17].

However, most studies reported so far are limited in discrete outcome and sample size. Therefore, we conducted a quantitative meta-analysis to clarify the prognostic and clinicopathological significance of lncRNA HOXA11-AS expression in patients with cancer.

## Materials and methods

### Literature collection

The present review was performed in accordance with the standard guidelines for meta-analysis of tumor marker prognostic studies [18, 19]. Two authors (Mu and Ai) independently used the following tools: PubMed, Embase, ISI Web of Science, and SCOPUS to obtain relevant articles [20] on prognostic and clinicopathological significance of HOXA11-AS in patients with any cancer. The last search date was December 12, 2017. The search strategy was: “HOXA11-ASor HOXA11 antisense RNA or HOXA11AS or HOXA-AS5” and “long noncoding RNA or lncRNA or noncoding RNA or RNA long noncoding” and “cancer or tumor or carcinoma or neopla* or malignan*”. We also retrieved articles from other sources, such as the reference lists of relevant articles.

### Study selection

The same two researchers (Mu and Ai) independently assessed all the included studies and extracted the data. Studies were considered eligible if they met the following inclusion criteria: (1) any type of human cancer was involved; (2) all tumors were diagnosed through pathological or histological examinations; (3) HOXA11-AS expression was measured in human tissue or plasma; (4) the patients had to be divided into two groups according to the expression level of HOXA11-AS; (5) the survival curve or sufficient relevant data were provided to obtain hazard ratios (HR) for survival rates and their 95% confidence intervals.

Studies were excluded if they met the following criteria: (1) they were letters, case reports, reviews, conference abstracts, etc.; (2) studies with insufficient data for estimating HR/OR and 95% CI; (3) animal studies, cellular level studies or molecular level studies of HOXA11-AS; (4) multiple published reports. When there were several reports concerning the same cohort we included the high quality and most recent publication in our meta-analysis. Any disagreement was resolved by the third party (Sun and Hu).

### Data extraction

Data extraction was repeated independently by the two researchers (Mu and Fan), and in the situation of a disagreement, a consensus was reached by a third researcher (Sun and Hu). For each study, the following characteristics of the individual research article were collected: author; year of publication; country of the population enrolled; sample size; study design; follow-up data; overall survival (OS); progression-free survival (PFS); survival analysis methodology; sample type, HOXA11-AS expression level; cut-off values; treatment information; HR values and their 95% confidence intervals; and relevant clinicopathological parameters such as age, gender, stages, histological differentiation, and lymph node metastasis. We extracted the reported HRs and their 95% confidence intervals (CIs) directly from the publication [21]. However, there were some HRs with 95% CIs that could not be directly obtained. In this case, we extracted necessary data from Kaplan–Meier Curves, and inputted the obtained survival rates at specified time points into the spreadsheet set up by Tierney et al. to calculate HRs and their 95% confidence intervals using the Engauge Digitizer version 9.8. If possible, we asked for original data directly from the authors of the relevant studies.

### Quality assessment

A quality assessment was independently performed in each of the included studies by two reviewers (Mu and Ai) using the Newcastle–Ottawa Quality Assessment Scale (NOS) [22]. This scale uses a star system (with a maximum of nine stars) to evaluate a study in three domains: selection of participants, comparability of study groups, and the ascertainment of outcomes of interest. NOS scores of ≥ 6 were assigned as high-quality studies. Any disagreement was resolved by the third party (Sun and Hu).

### Statistical analysis

Pooled hazard ratios (HRs) and their associated 95% confidence intervals (CIs) were used to analyze the prognostic role of HOXA11-AS expression in various solid tumors [23]. Pooled odds ratios (ORs) and their associated 95% CIs were used to analyze the association between HOXA11-AS expression and clinicopathological parameters.

Heterogeneity among included studies was checked by the χ^2^ based Q test and I^2^ test [24]. The fixed-effect model was used for analysis without any significant heterogeneity between studies (*p* > 0.10, I^2^ < 50%). Otherwise, the random-effects model was chosen. Subgroup analysis and meta regression were further performed to explore the source of heterogeneity. Influence analysis was performed to examine the effect of each study on the overall pooled results. All statistical tests were two sided and the significance level was set at 5%.

The Begg’s funnel plot was used to visually evaluate the publication bias of all studies included in our meta-analysis [25]. And then the Egger’s bias indicator test was performed for each of the pooled study groups. All analyses were carried out using STATA statistical software package version 14.0 (STATA, College Station, TX).

## Results

### Included studies

**Fig. 1:**
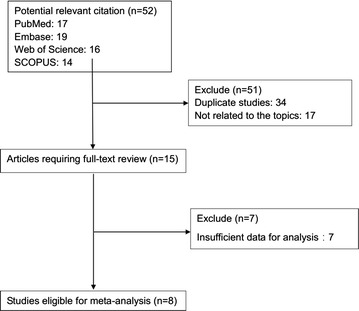
Flow diagram of selecting relevant published works regarding HOXA11-AS in cancer

As shown in Fig. 1, the initial search algorithm retrieved a total of 52 studies (PubMed = 17, Embase = 19, ISI Web of Science = 16, SCOPUS = 14). After excluding the duplicates (n = 34), and the studies not related to research topics (n = 17), the remaining studies (n = 15) were further reviewed by reading the full text. Additional studies were then excluded because they didn’t provide sufficient data for analysis (n = 7). Therefore, a total of eight studies [17, 26–32], including six on clinicopathological features, and eight on prognosis, were eligible for the final analysis.

### Study characteristics

A total of 1320 cases from eight included eligible studies with relevant clinical data were included in this meta-analysis (Table 1). In summary, (1) the sample sizes of these studies ranging from 45 to 463; (2) the year of publication ranges from 2016 to 2017; (3) six of these studies were conducted in China; (4) six types of carcinomas, including cervical cancer, gastric cancer, glioblastoma, glioma, non-small cell lung cancer, osteosarcoma and serous ovarian cancer, were involved in the enrolled studies. The lncRNA HOXA11-AS expression levels in these studies were mostly measured by quantitative real time PCR (qRT-PCR).

**Table 1:** Characteristics of studies included in the meta-analysis

Authors	Years	Region	Tumor type	Sample type	Sample size	Preoperative treatment	Tumor stage	Method of HOXA11-AS expression	Elevated HOXA11-AS	Cut-off value	Outcome measure	Survival analysis^a^	Method^b^	NOS score
Kim et al.	2016 [14, 17]	Korea	Cervical cancer	Tissues	92	No	FIGO I–III	qRT-PCR	Significantly higher (p < 0.05)	Tenfold	OS	U, M	1	8
Sun et al.	2016 [31]	China	Gastric cancer	Tissues	86	No	TNM I–IV	qRT-PCR	Significantly higher (p < 0.01)	Median	OS, PFS	U, M	1, 2	8
Wang et al.	2016 [26]	China	Glioblastoma	Tissues	89	N/A	N/A	N/A	Significantly higher (p < 0.01)	Median	OS	U, M	1	6
Chen et al.	2017 [9, 27]	China	Non-small cell lung cancer	Tissues	78	No	TNM I–III	qRT-PCR	Significantly higher (p < 0.05)	Median	OS	No	2	7
Cui et al.	2017 [16, 28]	China	Osteosarcoma	Tissues	51	No	TNM I–III	qRT-PCR	Significantly higher (p < 0.05)	Median	OS	No	1	6
Xu et al.	2017 [32]	China	Glioma	Tissues	45	N/A	TNM I–IV	qRT-PCR	Significantly higher (p < 0.01)	Median	OS	No	1	7
Yim et al.	2017 [29]	Korea	Serous ovarian cancer	Tissues	129	No	FIGO I–IV	qRT-PCR	Significantly higher (p < 0.05)	Fold change	OS, PFS	M	1	7
Zhang et al.	2017 [30]	TCGA	Lung adenocarcinoma	N/A	287	N/A	TNM I–IV	N/A	Significantly higher (p < 0.001)	N/A	OS	No	2	6
			Lungsquamous cell carcinoma	N/A	463	N/A	TNM I–IV	N/A	Significantly higher (p < 0.001)	N/A	OS	No	2	6

*N/A* not available, *TNM* tumor/node/metastasis, *FIGO* International Federation of gynecology and obstetrics, *qRT-PCR* quantitative reverse transcription PCR, *OS* overall survival, *PFS* progression free survival, *NOS* Newcastle–Ottawa Scale

^a^U denoted as univariate analysis; M denoted as multivariate analysis

^b^1 denoted as obtaining HRs directly from publications; 2 denoted as extracting HRs from Kaplan–Meier curves

### Association between HOXA11-AS and clinicopathological characteristics of cancers

We pooled all the clinicopathological data from these eligible studies to analyze the clinicopathological significance of HOXA11-AS expression level in cancers. As shown in Table 2, the meta-analytic results showed that the increased expression level of HOXA11-AS was significantly associated with more lymph node metastasis (OR = 2.06, 95% CI 1.31–3.25), and advanced tumor stage (OR = 4.217, 95% CI 2.595–6.853). Moreover, the OR of poor tumor differentiation in cancer patients with elevated HOXA11-AS expression level was 2.49 (95% CI 1.47–4.20, *p* = 0.001). However, none of the studies demonstrated significant association between HOXA11-AS expression level with patients’ age or gender (*p* = 0.101 and *p* = 0.845, respectively).

**Table 2:** Association between HOXA11-AS and clinicopathological characteristics of cancers

Clinicopathological parameters	Studies (n)	Patients (n)	OR (95% CI)	*p* value	Heterogeneity		
					I2 (%)	p	Model
Age (≥ 55 vs. < 55 years)	4	259	0.66 (0.40–1.08)	0.101	0	0.899	Fixed
Gender (male vs. female)	4	259	1.05 (0.64–1.72)	0.845	0	0.483	Fixed
Lymph node metastasis (yes vs. no)	4	384	2.06 (1.31–3.25)	0.002	47.1	0.129	Fixed
Tumor stage (III–IV vs. I–II)	6	480	4.22 (2.60–6.85)	< 0.001	0	0.993	Fixed
Tumor differentiation (poor vs. well)	3	292	2.49 (1.47–4.20)	0.001	0	0.648	Fixed

### Association between HOXA11-AS and survival in six types of cancers

**Fig. 2:**
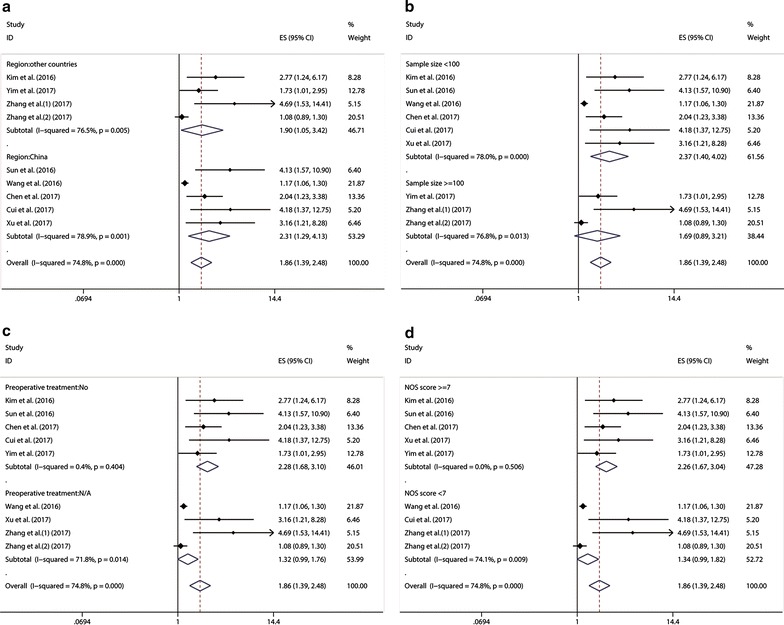
Meta-analysis of the association between elevated HOXA11-AS and OS in cancer by **a** region, **b** sample size, **c** preoperative treatment, **d** paper quality

Eight studies reported the overall survival (OS) of six types of cancer based on different HOXA11-AS expression levels in a total of 1320 patients. A significant association was found between elevated HOXA11-AS expression and poor OS in cancer patients (pooled HR = 1.86, 95% CI 1.39–2.48). Significant heterogeneity existed across the studies (*p* < 0.001; I^2^ = 74.8%). In order to explore the source of heterogeneity, subgroups were analyzed by factors of the region (China or other countries), sample size (more than 100 or fewer than 100), preoperative treatment (No or unclear), and paper quality (NOS scores ≥ 7 or < 7) (Fig. 2). There was no significant association between HOXA11-AS expression and OS in the subgroup of patients with unclear preoperative treatment, nor in the studies with more than 100 patients. However, heterogeneity significantly decreased in the subgroup of patients with no preoperative treatment, and studies of high paper quality (NOS scores ≥ 7). Meta-regression didn’t reveal *p* values less than 0.05 in the above four covariates (Table 3). According to sensitivity analysis, we re-evaluate the prognostic role of HOXA11-AS expression in OS for cancer patients after exclude these two outlier studies [26, 30], and the combined HR for the remaining studies is 2.45 (95% CI 1.85–3.25), without any significant heterogeneity across the studies (*p* = 0.445; I^2^ = 0).

**Table 3:** Subgroup analysis and meta regression of pooled HRs for OS in cancer patients with increased HOXA11-AS expression

Subgroup analysis	No. of studies	No. of patients	Pooled HR (95% CI)	Meta regression (p value)	Heterogeneity	
					I2	p value
Region						
China	5	349	2.31 (1.29–4.13)		78.9%	0.001
Other	3	971	1.90 (1.05–3.42)	0.677	76.5%	0.005
Sample size						
≥ 100	2	879	1.69 (0.89–3.21)		76.8%	0.013
< 100	6	441	2.37 (1.40–4.02)	0.469	78%	< 0.001
Preoperative treatment						
No	5	436	2.28 (1.68–3.10)		0.4%	0.404
Unclear	3	884	1.32 (0.99–1.76)	0.187	71.8%	0.014
NOS score						
≥ 7	5	430	2.26 (1.67–2.98)		0	0.506
< 7	3	890	1.77 (1.32–2.37)	0.238	74.1%	0.009

Using Cox multivariate analysis in three studies from Kim et al. [14, 17], Sun et al. [31], and Wang et al. [26], we found that elevated HOXA11-AS expression level was an independent prognostic factor of OS for cancer patients (pooled HR = 1.184, 95% CI 1.050–1.335, *p* = 0.006), without significant heterogeneity among studies (*p* = 0.07; I^2^ = 62.4%).

**Fig. 3:**
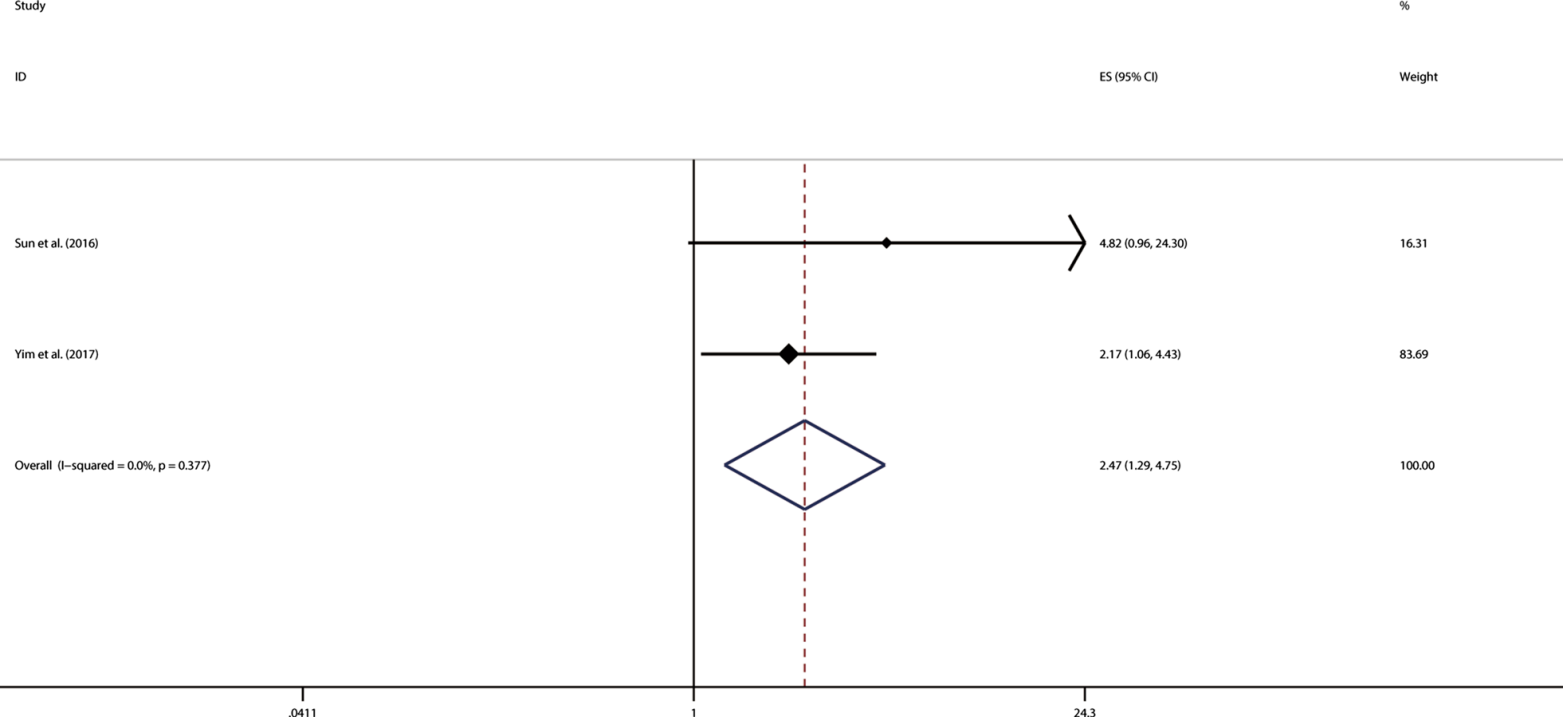
Meta-analysis of the association between elevated HOXA11-AS and PFS in cancer

Two studies [29, 31] with 215 cancer patients are pooled to estimate the prognostic role of HOXA11-AS expression in PFS for cancer patients. The results showed that elevated HOXA11-AS expression level was significantly associated with shorter PFS for cancer patients (pooled HR = 2.47, 95% CI 1.29–4.75) without significant heterogeneity (*p* = 0.377; I^2^ = 0) (Fig. 3). Because these two studies used cox multivariate analysis, we can conclude that elevated HOXA11-AS expression level is an independent prognostic factor of PFS for cancer patients.

### Publication bias

**Fig. 4:**
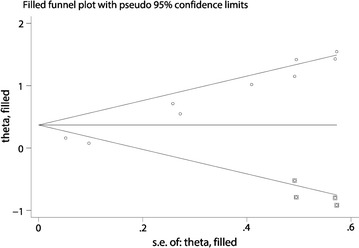
Trim and fill analysis of the eligible studies for the present meta-analysis

Begg’s test and Egger’s linear regression test were conducted to evaluate publication bias. The funnel plot showed significant asymmetry (data not shown), and Begg’s test and Egger’s linear regression test also proved that there was significant publication bias (*p* = 0.009 and *p* < 0.001, respectively). Using trim and fill analysis, we found that four studies evaluating the prognostic role of HOXA11-AS expression in OS for cancer patients remained unpublished (Fig. 4). The filled meta-analytic results for OS (HR = 1.45, 95% CI 1.09–1.91) supported our original results.

### Influence analysis

**Fig. 5:**
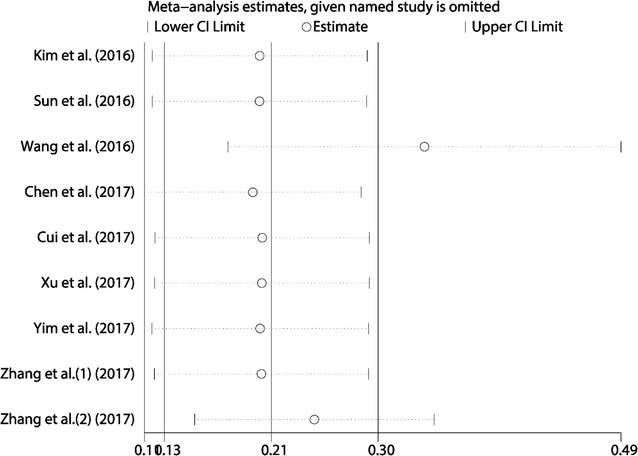
Influence analysis of the studies enrolled in the present meta-analysis

As shown in Fig. 5, the influence analysis identified that there were two cohorts from Wang et al. [26] and Zhang et al. [30] influencing the results greatly. The 95% CIs of pooled HRs and heterogeneity changed notably after excluding these two studies. However, the list of pooled HRs and 95% CIs after excluding single study one by one indicated robustness of our results, in which all pooled HRs and 95% CIs were above the null hypothesis of 1 (data not shown).

## Discussion

Recently, substantial evidence has demonstrated that lncRNAs are important regulatory molecules in tumorigenesis. It has been reported that lncRNA FAL1 induces epithelial-to-mesenchymal transition (EMT) in non-small cell lung cancer (NSCLC) [33]. In gastric cancer, lncRNA H19 promotes cell proliferation and inhibits cell apoptosis [34]. Li et al. [35] showed that LncRNA HOTAIR enhanced invasion and metastasis of breast cancer. Moreover, lncRNA HOXA11-AS has been found to induce tumor progression and stemness maintenance in cervical cancer [14]. Many lncRNAs are identified in various types of cancers, and their dysregulated expression is correlated with tumor progression and patients’ survival, thus making them promising markers for the diagnosis and prognosis. For example, the lncRNAs TUG1 [36], UCA1 [37], SPRY4-IT1 [38], HULC [9], HOTTIP [39], H19 [40] and HOTAIR [41] were found to be novel promising biomarkers for poor prognosis in human cancers.

A previous meta-analysis combining seven studies has reported that high lncRNA HOXA11-AS expression was a significant indicator for poor OS and PFS from a total of 608 individuals [42]. Here we conducted independently a meta-analysis to investigate whether lncRNA HOXA11-AS can be served as a prognostic biomarker in human cancers. The present comprehensive meta-analysis pooled a total of nine independent cohorts with 1320 cancer patients, and the results indicated that high HOXA11-AS expression was a prognostic risk factor for OS in cancer patients (HR = 1.86, 95% CI 1.39–2.48).

Subgroup analysis, meta regression analysis and sensitivity analysis were performed due to significant heterogeneity across these studies. The results of subgroup analysis suggested that sample size (more than 100 or fewer than 100) and preoperative treatment (No or unclear) altered the significance of HOXA11-AS prognostic role in OS (HR = 1.69, 95% CI 0.89–3.21 vs. HR = 2.37, 95% CI 1.40–4.02; and HR = 2.28, 95% CI 1.68–3.10 vs. HR = 1.32, 95% CI 0.99–1.76, respectively). However, meta regression analysis failed to identify the source of the significant heterogeneity in these above four covariates (*p* > 0.05). Besides, the influence analysis indicated that there were two cohorts from Wang et al. [26] and Zhang et al. [30] impacting the pooled HR and its 95% CI apparently. After excluding these two outlier cohorts above, the pooled results were in line with the full meta-analytic results (pooled HR = 2.45, 95% CI 1.85–3.25) without any significant heterogeneity across the remaining studies (*p* = 0.445; I^2^ = 0). The reason why the study from Wang et al. [26] caused obvious heterogeneity might owe to the much younger age of patients suffering from osteosarcoma or the different origin of the tumor cells. As for the cohorts from Zhang et al. [30], data were from the Cancer Genome Atlas (TCGA), lacking sufficient clinicopathological information for patients’ normalization. However, the results of the cohort from Zhang et al. [30] was in accordance with the full meta-analytic results. We inferred that the biological types of carcinoma and different patients’ basic status might have notable influence. Moreover, the prognostic significance of HOXA11-AS in PFS was evaluated in two studies with 215 patients, and the results indicated that patients with high HOXA11-AS expression were possible to have significantly shorter PFS (pooled HR = 2.47, 95% CI 1.29–4.75) with no significant heterogeneity. Furthermore, by combining HRs from Cox multivariate analyses, we found that HOXA11-AS was an independent prognostic factor of OS and PFS in cancer patients.

Importantly, our analysis revealed that elevated expression level of HOXA11-AS was significantly related to advanced TNM stage and poor tumor differentiation. However, there was no significant association between HOXA11-AS expression and age or gender of patients. In addition, two studies from Sun et al. and Cui et al. suggested that elevated expression level of HOXA11-AS was significantly related to more distant metastasis (OR = 5.782, 95% CI 1.579–21.173). However, the study from Kim et al. showed the insignificant association between HOXA11-AS expression and lymphovascular invasion (OR = 1.556, 95% CI 0.680–3.558), or recurrence (OR = 1.688, 95% CI 0.661–4.309).

Mechanisms underlying the regulatory role of HOXA11-AS in tumorigenesis and tumor progression have been extensively investigated in various types of cancer. Kim et al. found that HOXA11-AS promoted cancer stem cells (CSCs) self-renewal and EMT in cervical cancer cells through regulating the expression of SOX2, Oct-4, Nanog, E-cadherin, β-catenin, and Vimentin [14]. Sun et al. [31] revealed that HOXA11-AS served as a critical effector in gastric cancer tumorigenesis and progression via HOXA11-AS/miR-1297/EZH2 crosstalk. Study from Chen et al. indicated that HOXA11-AS promoted EMT in NSCLC through interacting with EZH2 and DNMT1 and repressing miR-200b expression [27]. In osteosarcoma, HOXA11-AS functions as a competing endogenous RNA and regulates ROCK1 expression by sponging miR-124-3p, thus promoting cell proliferation and metastasis [28]. A better understanding of the functions of HOXA11-AS in human cancer may help the development of new prognostic and therapeutic strategies.

Recent studies have reported dysregulated HOXA11-AS expression in multiple types of cancers, and demonstrated the underlying mechanisms on lncRNA HOXA11-AS regulating the malignant phenotypes of cancer cells. The present meta-analysis indicated that HOXA11-AS, an oncogenic lncRNA, was a promising biomarkers for prognosis estimation. However, there are limited studies on the regulatory role of HOXA11-AS in the conversion from precancerous lesions to malignancies. For now, it’s quite difficult to figure out the functional importance of HOXA11-AS expression during tumorigenesis. Therefore, more relevant studies are warranted to fill the gap and promote the clinical application of HOXA11-AS as early detectable indicator.

Compared to other biomarkers for cancer, lncRNA HOXA11-AS has been growing to be a promising prognostic biomarkers for reasons as followed: (1) our study demonstrated that overexpressed HOXA11-AS was an independent unfavorable prognostic factor of OS and PFS in cancer patients; (2) many studies revealed that lncRNAs reflected more tumor biological characteristics through interacting with protein coding genes and miRNAs, thus affecting cell growth, apoptosis, and metastasis, etc.

However, the current meta-analysis had some limitations calling for cautious interpretation of the results. First, only eight studies published in full-text were pooled for our meta-analysis, and the data of three cohorts were from databases online (such as TCGA and CCGA). Second, the cut-off value of high and low HOXA11-AS expression level were different among studies, although most of them were set to median. Third, HRs of five cohorts could not be directly obtained from the publications. Moreover, calculating HRs and corresponding 95% CIs through survival curves might not be precise enough. Forth, most of the included studies reported positive results so that our results might overestimate the prognostic significance of HOXA11-AS in cancer to some degree. In case of the significant publication bias, we used trim and fill analysis, and only found another four studies unpublished. However, the filled meta-analytic results supported our original results. Finally, cancer is a complicate and heterogeneous disease. It’s quiet difficult to extrapolate conclusions on the biologic role of HOXA11-AS during tumorigenesis, especially when we take various solid tumors in account. And grading, staging and the investigation of precancerous lesions are important aspects to define the regulatory role of HOXA11-AS in the early or late stages of cancer. Therefore, larger-scale, multi-center, and high-quality studies are warranted to validate our findings.

## Conclusions

Our study found that HOXA11-AS overexpression might be a convinced unfavorable prognostic factor helpful for the clinical decision-making process. Moreover, the expression level of HOXA11-AS was associated with clinicopathological parameters, including TNM stage, and lymph node metastasis. With rapid development of high throughput sequencing technology, lncRNA HOXA11-AS may become a novel prognostic biomarker and therapeutic targets for cancer. In the future, more relevant studies are warranted to investigate the role of HOXA11-AS in human cancer.

## Abbreviations

ncRNA: noncoding RNA
lncRNA: long noncoding RNA
HOXA11-AS: the homeobox A11 antisense lncRNA
EMT: epithelial–mesenchymal transition
HR: hazard ratios
OR: odds ratios
OS: overall survival
PFS: progression-free survival
95% CIs: 95% confidence intervals
NOS: The Newcastle–Ottawa Quality Assessment Scale
qRT-PCR: quantitative real time PCR
NSCLC: non-small cell lung cancer
TCGA: The Cancer Genome Atlas
CCGA: The China Cancer Genome Atlas
CSC: cancer stem cells

## References

[1] Siegel RL, Miller KD, Jemal A (2017). Cancer statistics, 2017. CA Cancer J Clin.

[2] Chen W, Zheng R, Baade PD, Zhang S, Zeng H, Bray F, Jemal A, Yu XQ, He J (2016). Cancer statistics in China, 2015. CA Cancer J Clin.

[3] Guttman M, Rinn JL (2012). Modular regulatory principles of large non-coding RNAs. Nature.

[4] Schmitt AM, Chang HY (2016). Long noncoding RNAs in cancer pathways. Cancer Cell.

[5] Moran VA, Perera RJ, Khalil AM (2012). Emerging functional and mechanistic paradigms of mammalian long non-coding RNAs. Nucleic Acids Res.

[6] Cheetham SW, Gruhl F, Mattick JS, Dinger ME (2013). Long noncoding RNAs and the genetics of cancer. Br J Cancer.

[7] Huang T, Alvarez A, Hu B, Cheng SY (2013). Noncoding RNAs in cancer and cancer stem cells. Chin J Cancer.

[8] Yang G, Lu X, Yuan L (2014). LncRNA: a link between RNA and cancer. Biochem Biophys Acta.

[9] Chen X, Lun L, Hou H, Tian R, Zhang H, Zhang Y (2017). The value of lncRNA HULC as a prognostic factor for survival of cancer outcome: a meta-analysis. Cell Physiol Biochem.

[10] De Clara E, Gourvest M, Ma H, Vergez F, Tosolini M, Dejean S, Demur C, Delabesse E, Recher C, Touriol C et al. Long noncoding RNA expression profile in cytogenetically normal acute myeloid leukemia identifies a distinct signature and a new biomarker in NPM1-mutated patients. Haematologica. 2017;102(10):1718–26. 10.3324/haematol.2017.171645 PMC5622856 28679652

[11] Wang W, He X, Zheng Z, Ma X, Hu X, Wu D, Wang M (2017). Serum HOTAIR as a novel diagnostic biomarker for esophageal squamous cell carcinoma. Mol Cancer.

[12] Richards EJ, Permuth-Wey J, Li Y, Chen YA, Coppola D, Reid BM, Lin H-Y, Teer JK, Berchuck A, Birrer MJ (2015). A functional variant in HOXA11-AS, a novel long non-coding RNA, inhibits the oncogenic phenotype of epithelial ovarian cancer. Oncotarget.

[13] Li W, Jia G, Qu Y, Du Q, Liu B, Liu B (2017). Long non-coding RNA (LncRNA) HOXA11-AS promotes breast cancer invasion and metastasis by regulating epithelial–mesenchymal transition. Med Sci Monit.

[14] Kim HJ, Lee JY, Nam EJ, Park SA, Kim S, Kim SW, Kim YT (2016). Upregulation of long noncoding RNA HOXA11 antisense promotes tumor progression and stemness maintenance of cervical cancer cells. Gynecol Oncol.

[15] Liu Z, Chen Z, Fan R, Jiang B, Chen X, Chen Q, Nie F, Lu K, Sun M (2017). Over-expressed long noncoding RNA HOXA11-AS promotes cell cycle progression and metastasis in gastric cancer. Mol Cancer.

[16] Cui Y, Yi L, Zhao JZ, Jiang YG (2017). Long noncoding RNA HOXA11-AS functions as miRNA sponge to promote the glioma tumorigenesis through targeting miR-140-5p. DNA Cell Biol.

[17] Kim HJ, Eoh KJ, Kim LK, Nam EJ, Yoon SO, Kim KH, Lee JK, Kim SW, Kim YT (2016). The long noncoding RNA HOXA11 antisense induces tumor progression and stemness maintenance in cervical cancer. Oncotarget.

[18] McShane LM, Altman DG, Sauerbrei W, Taube SE, Gion M, Clark GM (2005). Reporting recommendations for tumor MARKer prognostic studies (REMARK). Nat Clin Pract Oncol.

[19] Altman DG, McShane LM, Sauerbrei W, Taube SE (2012). Reporting recommendations for tumor marker prognostic studies (REMARK): explanation and elaboration. PLoS Med.

[20] Dickersin K, Scherer R, Lefebvre C (1994). Identifying relevant studies for systematic reviews. BMJ.

[21] Parmar MK, Torri V, Stewart L (1999). Extracting summary statistics to perform meta-analyses of the published literature for survival endpoints. Stat Med.

[22] Wells GA, Shea B, O’Connell D, Peterson J, Welch V, Losos M, Tugwell P. The Newcastle-Ottawa Scale (NOS) for assessing the quality of nonrandomized studies in meta-analyses. Ottawa Hospital Research Institute; 2013. p. 1–4.

[23] Moher D, Liberati A, Tetzlaff J, Altman DG (2009). Preferred reporting items for systematic reviews and meta-analyses: the PRISMA statement. PLoS Med.

[24] Higgins JP, Thompson SG, Deeks JJ, Altman DG (2003). Measuring inconsistency in meta-analyses. BMJ.

[25] Stuck AE, Rubenstein LZ, Wieland D, Vandenbroucke JP, Irwig L, Macaskill P, Berry G, Glasziou P, Seagroatt V, Stratton I (1997). Bias in meta-analysis detected by a simple, graphical test. BMJ.

[26] Wang Q, Zhang J, Liu Y, Zhang W, Zhou J, Duan R, Pu P, Kang C, Han L (2016). A novel cell cycle-associated lncRNA, HOXA11-AS, is transcribed from the 5-prime end of the HOXA transcript and is a biomarker of progression in glioma. Cancer Lett.

[27] Chen JH, Zhou LY, Xu S, Zheng YL, Wan YF, Hu CP (2017). Overexpression of lncRNA HOXA11-AS promotes cell epithelial–mesenchymal transition by repressing miR-200b in non-small cell lung cancer. Cancer Cell Int.

[28] Cui M, Wang J, Li Q, Zhang J, Jia J, Zhan X (2017). Long non-coding RNA HOXA11-AS functions as a competing endogenous RNA to regulate ROCK1 expression by sponging miR-124-3p in osteosarcoma. Biomed Pharmacother.

[29] Yim GW, Kim HJ, Kim LK, Kim SW, Kim S, Nam EJ, Kim YT (2017). Long non-coding RNA HOXA11 antisense promotes cell proliferation and invasion and predicts patient prognosis in serous ovarian cancer. Cancer Res Treat.

[30] Zhang Y, Chen WJ, Gan TQ, Zhang XL, Xie ZC, Ye ZH, Deng Y, Wang ZF, Cai KT, Li SK (2017). Clinical significance and effect of lncRNA HOXA11-AS in NSCLC: a study based on bioinformatics, in vitro and in vivo verification. Sci Rep.

[31] Sun M, Nie F, Wang Y, Zhang Z, Hou J, He D, Xie M, Xu L, De W, Wang Z (2016). LncRNA HOXA11-AS promotes proliferation and invasion of gastric cancer by scaffolding the chromatin modification factors PRC2, LSD1, and DNMT1. Can Res.

[32] Xu C, He T, Li Z, Liu H, Ding B (2017). Regulation of HOXA11-AS/miR-214-3p/EZH2 axis on the growth, migration and invasion of glioma cells. Biomed Pharmacother.

[33] Pan C, Yao G, Liu B, Ma T, Xia Y, Wei K, Wang J, Xu J, Chen L, Chen Y (2017). Long noncoding RNA FAL1 promotes cell proliferation, invasion and epithelial–mesenchymal transition through the PTEN/AKT signaling axis in non-small cell lung cancer. Cell Physiol Biochem.

[34] Yan J, Zhang Y, She Q, Li X, Peng L, Wang X, Liu S, Shen X, Zhang W, Dong Y (2017). Long noncoding RNA H19/miR-675 axis promotes gastric cancer via FADD/Caspase 8/Caspase 3 signaling pathway. Cell Physiol Biochem.

[35] Li M, Li X, Zhuang Y, Flemington EK, Zhen L, Shan B (2017). Induction of a novel isoform of the lncRNA HOTAIR in Claudin-low breast cancer cells attached to extracellular matrix. Mol Oncol.

[36] Liu J, Lin J, Li Y, Zhang Y, Chen X (2017). Prognostic role of lncRNA TUG1 for cancer outcome: evidence from 840 cancer patients. Oncotarget.

[37] Wang X, Peng F, Cheng L, Yang G, Zhang D, Liu J, Chen X, Zhao S (2017). Prognostic and clinicopathological role of long non-coding RNA UCA1 in various carcinomas. Mol Cancer.

[38] Wang M, Dong X, Feng Y, Sun H, Shan N, Lu T (2017). Prognostic role of the long non-coding RNA, SPRY4 Intronic Transcript 1, in patients with cancer: a meta-analysis. Oncotarget.

[39] Jin N, Yang LY, Xu ZP (2017). Long non-coding RNA HOTTIP is able to predict poor prognosis in various neoplasms: a meta-analysis. Mol Clin Oncol.

[40] Chu M, Yuan W, Wu S, Wang Z, Mao L, Tian T, Lu Y, Zhu B, Yang Y, Wang B (2016). Quantitative assessment of polymorphisms in H19 lncRNA and cancer risk: a meta-analysis of 13,392 cases and 18,893 controls. Oncotarget.

[41] Serghiou S, Kyriakopoulou A, Ioannidis JP (2016). Long noncoding RNAs as novel predictors of survival in human cancer: a systematic review and meta-analysis. Mol Cancer.

[42] Li N, Yang M, Shi K, Li W (2017). Long non-coding RNA HOXA11-AS in human cancer: a meta-analysis. Clin Chim Acta.

